# A novel machine learning model to predict respiratory failure and invasive mechanical ventilation in critically ill patients suffering from COVID-19

**DOI:** 10.1038/s41598-022-14758-x

**Published:** 2022-06-22

**Authors:** Itai Bendavid, Liran Statlender, Leonid Shvartser, Shmuel Teppler, Roy Azullay, Rotem Sapir, Pierre Singer

**Affiliations:** 1 Department of General Intensive Care and Institute for Nutrition Research, Rabin Medical Center, Beilinson Hospital, 39 Jabotinsky St, Petah Tikva, Israel; 2 TSG IT Advanced Systems Ltd., Tel Aviv, Israel

**Keywords:** Diseases, Respiratory tract diseases, Respiratory distress syndrome

## Abstract

In hypoxemic patients at risk for developing respiratory failure, the decision to initiate invasive mechanical ventilation (IMV) may be extremely difficult, even more so among patients suffering from COVID-19. Delayed recognition of respiratory failure may translate into poor outcomes, emphasizing the need for stronger predictive models for IMV necessity. We developed a two-step model; the first step was to train a machine learning predictive model on a large dataset of non-COVID-19 critically ill hypoxemic patients from the United States (MIMIC-III). The second step was to apply transfer learning and adapt the model to a smaller COVID-19 cohort. An XGBoost algorithm was trained on data from the MIMIC-III database to predict if a patient would require IMV within the next 6, 12, 18 or 24 h. Patients’ datasets were used to construct the model as time series of dynamic measurements and laboratory results obtained during the previous 6 h with additional static variables, applying a sliding time-window once every hour. We validated the adaptation algorithm on a cohort of 1061 COVID-19 patients from a single center in Israel, of whom 160 later deteriorated and required IMV. The new XGBoost model for the prediction of the IMV onset was trained and tested on MIMIC-III data and proved to be predictive, with an AUC of 0.83 on a shortened set of features, excluding the clinician’s settings, and an AUC of 0.91 when the clinician settings were included. Applying these models “as is” (no adaptation applied) on the dataset of COVID-19 patients degraded the prediction results to AUCs of 0.78 and 0.80, without and with the clinician’s settings, respectively. Applying the adaptation on the COVID-19 dataset increased the prediction power to an AUC of 0.94 and 0.97, respectively. Good AUC results get worse with low overall precision. We show that precision of the prediction increased as prediction probability was higher. Our model was successfully trained on a specific dataset, and after adaptation it showed promise in predicting outcome on a completely different dataset. This two-step model successfully predicted the need for invasive mechanical ventilation 6, 12, 18 or 24 h in advance in both general ICU population and COVID-19 patients. Using the prediction probability as an indicator of the precision carries the potential to aid the decision-making process in patients with hypoxemic respiratory failure despite the low overall precision.

## Introduction

Acute hypoxemic respiratory failure is a devastating condition, associated with very high morbidity and mortality rates^1^. It is a heterogeneous state with many known causes, potentially leading to lung dysfunction and respiratory muscle pump failure. Respiratory support may be noninvasive, mainly oxygen support, including high flow systems and noninvasive ventilation (NIV), or invasive mechanical ventilation (IMV) via tracheal intubation. Delayed recognition of respiratory failure and prolonged use of high flow oxygen therapy^2^ or NIV^3^ may lead to increased mortality. As decisions to initiate IMV are very often not clear cut, it is imperative to develop decision-support tools that may help assess which patients are more prone to fail under noninvasive support. These decisions are more relevant than ever when treating COVID-19 patients in respiratory failure, in whom NIV and high flow oxygen therapy are used in around 15% of these patients' population^4^.

We devised a new model to improve the prediction of hypoxemic respiratory failure based on the existing, large dataset of the Medical Information Mart for Intensive Care (MIMIC-III). Although acute respiratory failure has some similarity to respiratory failure due to COVID-19, patients' reactions and morbidities are different, thus models trained on pre-COVID-19 population might not work well for prediction of the COVID-19 respiratory failure. Training the models only on COVID-19 population in a single hospital is also not adequate since the cohort size is rather small. Therefore, we chose two-step solution; first to train a machine learning (ML) predictive model on a large dataset of pre-COVID-19 general population, then adapt the model to the smaller COVID-19 cohort.

## Methods

### MIMIC-III database

**Figure 1:**
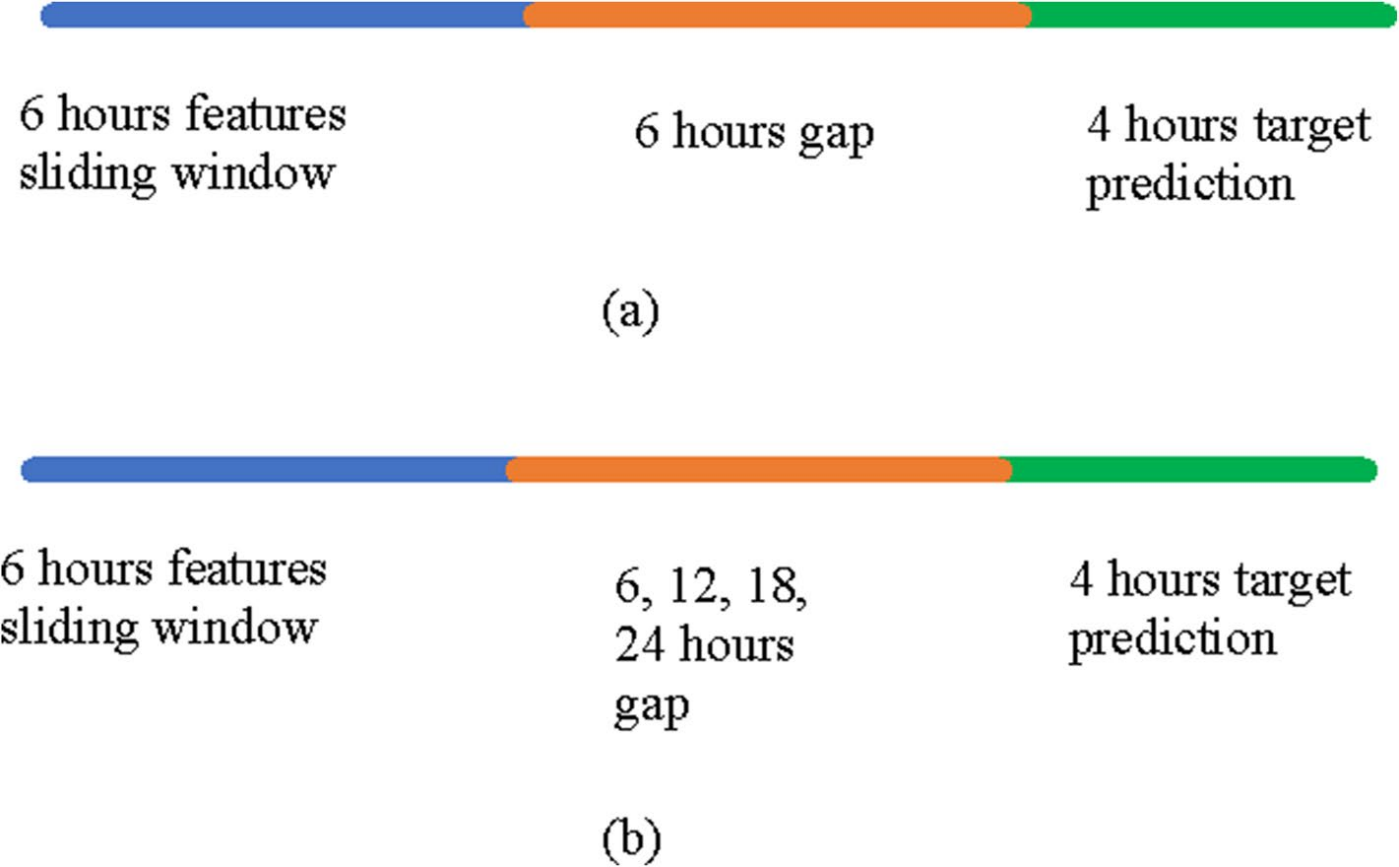
The sliding window prediction schemes. (**a**) In the first prediction window, the point of reference is at the end of the 6-h features sliding window. The model predicted the onset of invasive mechanical ventilation at the 4-h target prediction period while ignoring the six hours gap period. (**b**) A widened sliding window prediction scheme. This is a scheme similar to (**a**) but the gap period here varies between 6 to 24 h for the prediction of invasive mechanical ventilation.

The database comprises detailed clinical information for over 60,000 stays in ICUs at the Beth Israel Deaconess Medical Center in Boston, Massachusetts, collected as part of routine clinical care^5^. Patients' records are anonymized and readily available for researchers. The MIMIC extract provides standardized data processing functions, including unit conversion and outlier detection while preserving the time series nature of clinical data^6^. Values were mean centered and scaled to univariance, then missing data was imputed using a variant of the “Simple Imputation” scheme, in which each variable is represented via a mask (mask = 1 if the value is present at this time step, mask = 0 otherwise), the imputed variable and the time since the last observation of this feature. Several predictive algorithms are already implemented by the authors of MIMIC extract^6^, including mortality, length-of-stay^7^ and application of IMV. Application of IMV consisted of four predefined states: Onset, Stay On, Wean and Stay Off^8^. To make clinically meaningful predictions, MIMIC extract aggregates outputs as a sliding window with the size of 6 h as input features, then a prediction algorithm is applied to predict four possible interventions within a 4-h prediction window, offset by the input window by a 6-h gap window (Fig. 1a). The dataset was originally analyzed using logistic regression, random forest, convolutional neural network and long short-term memory^6^. We took these problems with the same data used in the past^6^ and solved them using XGBoost as it had been shown to outperform the aforementioned methods for all the tasks^9,10^.

The most important question for COVID-19 patients was to predict onset of IMV, therefore we reconstructed the model for onset prediction only. We focused on the cohort of acute hypoxemic respiratory failure patients, as it is the hallmark of respiratory failure in COVID-19 patients.

### Study design

IMV Onset prediction models were developed and tested using the MIMIC-III data. The models were validated on patients from Rabin medical center. Since models trained on non-COVID-19 MIMIC-III data may not be appropriate for the prediction of IMV onset in COVID-19 patients, a method that adapted the MIMIC-III model for COVID-19 patients was developed and evaluated on MIMIC-III data and validated on the Rabin COVID-19 data.

Patients’ datasets used to construct the model were time series of dynamic measurements and lab results, as well as additional static variables, while applying a sliding time-window once every hour. The datasets had 6-h sliding intervals, but only if in the first 6-h interval and in the following defined gap (6-h gap, or 12, 18, 24) IMV was not performed. IMV onset was detected for the 4 h after the gap, defined as positive (IMV in this window) or negative (no IMV in the window). The datasets were randomly divided into training (80%) and testing (20%). Some other important implementation details are presented in Supplement 1.

### Patients

Only hypoxemic patients in the MIMIC-III cohort were taken. According to MIMIC extract default parameters (intrinsic to the database): age > 15, minimal length of stay of 12 h, maximal length of stay of 10 days. The local study cohort comprised systems, staff reports and laboratory tests. Hypoxemia was defined as at least one of the following: PaO_2_ lower than 60 mmHg or SpO_2_ lower than 90%^11^. The Rabin hospital data tables were transformed to MIMIC-III tables, to allow use of the MIMIC extract pipeline. The data items describing patient demographics, admissions, vital signs, laboratory measurements, treatments and more are detailed in Supplement 1. We mapped the Rabin data for hypoxemic patients, only to the exact features of MIMIC-III, then we aligned the measurement units. There are features found at MIMIC-III yet not found on local data, and vice versa. We also modified these files to extract sets of variables that were best suited for our study and adjusted the custom outlier detection thresholds. Finally, we built the “Rabin-extract” pipeline, transforming all patients admitted to Rabin medical center between March 2020 and February 2021, that stayed in the hospital for more than 48 h (Rabin) and were diagnosed with COVID-19 (i.e., tested positive for the SARS-CoV-2 virus).

The trial was approved by the institutional review board of Rabin medical center. Consent was waived as this was an analysis of existing anonymized data. All experiments were performed in accordance with relevant guidelines and regulations.

### Data sources

The list of all the variables (Rabin mapping) participating in this study is presented in Supplement 2. The variables were continuously measured and stored in the electronic health record. While some of the variables were static, such as age or sex, most variables were dynamic, with periodic measurements from monitoring systems and labs. The relevant parts of the database were reformatted to the HDF5 file format, deemed more suitable for ML processing.

### Bias

A potential source of bias was the rather small cohort of COVID-19 patients in the Rabin database. To mitigate this bias, we chose a two-step solution: first to train the ML predictive model on a larger dataset of non-COVID-19 population, then to apply transfer learning and adapt the model to the smaller COVID-19 cohort.

### Machine learning and statistical methods

For the first step, the XGBoost algorithm^12^ was trained to answer only the question whether a patient not currently under invasive mechanical ventilation, will be invasively ventilated after the nearest 6, 12, 18, 24 h (Fig. 1b). XGBoost is a decision tree-based ensemble ML algorithm that uses a gradient boosting framework. XGBoost is considered the current strongest not-deep-learning ML algorithm, generally outperforming deep learning on moderate-size sets of data^9,10^.

For the adaptation, a dedicated transfer learning algorithm tailored for XGBoost was developed, as to preserve the predictive strength of the model trained on the source domain while applied on the target domain. The adaptation process used a portion of the target domain patients for fitting the source domain model to the target domain, and then test it on the complementary part of the target domain, i.e., remaining patients. The adaptation process is further described in Supplement 3. The IMV Onset prediction was trained and tested according to the scheme described above for 6-h gap. The results of evaluation of the algorithm obtained on MIMIC-III data are presented in Supplement 4. The code and the data to run this procedure and to evaluate it are available at https://github.com/lshvartser1959/TSG-ICU.

For the prediction of respiratory deterioration of hypoxemic COVID-19 patients, we used two methods of training and testing. The first method was a "one-step" approach: to train the model on Rabin COVID-19 data, in which we randomly split the data to 70% for training and the remaining 30% for testing. We took the structure of the algorithm, the pipeline, and the imputation method from our MIMIC-III preparations, using only features found relevant by feature importance analysis on MIMIC-III. The second method was a "two-step" approach: to train and test the model on MIMIC-III data, then apply the adaptation algorithm on the Rabin COVID-19 data. Training/testing/validation on MIMIC-III data were distributed 80/19.6/0.4%.

**Figure 2:**
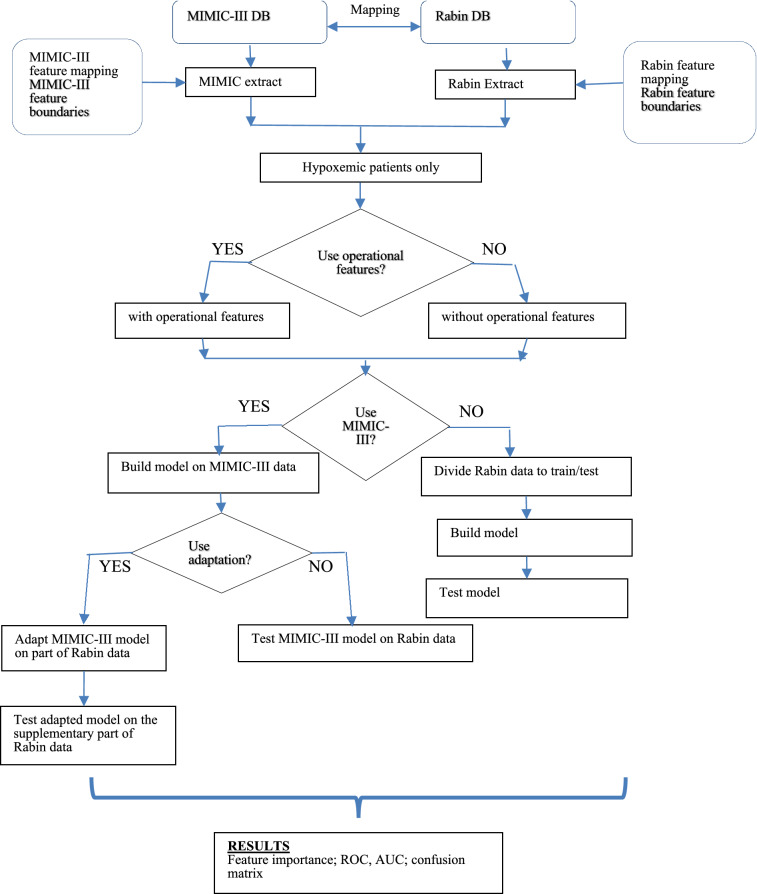
Processing flow diagram. Model training and testing is done in two configurations: with and without the operational features. Model for Rabin data may be pre-trained on MIMIC-III or without it. Finally, the model trained on MIMIC-III could be adapted to the Rabin data or tested as is**.** MIMIC-III: medical information mart for intensive care III. DB: database. ROC: receiver operator characteristic. AUC: area under the curve.

To map data for hypoxemic patients from both MIMIC-III and Rabin COVID-19 datasets, we trained models on both the original MIMIC extract mapping of features and the mapping that would have the ability to run on Rabin data (i.e., common features only, for both MIMIC-III and Rabin data). Features that were set by clinicians, mainly ventilator settings, were designated as operational features, as opposed to features from staff reports, monitoring systems and labs. Patients were randomly divided into groups of training and testing, to provide control for confounding variable, with no cross-over between groups. The aim of this adaptive two-step method was to mitigate the difference in progression towards respiratory failure and need for IMV between COVID-19 and non-COVID-19 patients. Missing data, including loss to follow-up, were imputed using a variant of the "simple imputation" scheme, in which each variable was represented via a mask and the time since the last measurement, as described earlier. Training / testing split was done in random 30 times for sensitivity analysis. The framework of the study and the data flow are presented in Fig. 2. Setups, training and testing options used in our experiments correspondingly on MIMIC-III and Rabin data are presented in Supplement 5.

### Feature importance

Each feature was given importance according to its relative contribution to the prediction of mechanical ventilation, as derived from the XGBoost model. Feature importance for 6-h gap in all MIMIC-III and Rabin configurations were examined. To estimate the influence of the features to classification we used the feature importance algorithm of XGBoost.

As previously mentioned, the XGBoost algorithm^12^ initially builds a decision tree to minimize losses in the prediction on the training set, in which outcomes are already known. It then gradually adds additional decision trees, to minimize residual losses using the Newton–Raphson method. The result is a model built as a sequence of decision trees. For classification purposes, every decision tree was built by choosing a sequence of features for splitting as well as thresholds for optimal classification of examples. Feature importance in XGBoost is defined as the number of times a variable is selected for splitting, weighted by the squared improvement of the model as a result of each split^13^. We chose the model's hyperparameters by random search on a validation set^14^. One of the tuned parameters was *scale_pos_weight*, ideal for work with unbalanced data by giving more weight to minor classes during calculation of the stochastic gradient.

Because of the sparse structure of data and the different rate of measurements, an imputation algorithm was employed. The imputation algorithm transformed data to a constant rate (1 h) and produced three features for each measured item^6^: mean value per hour, 0 or 1 mask for all measurements within 1 h, and time since last measurement that reflects the measurement’s accuracy- the longer the time since the last measurement, the lower the accuracy. The XGBoost algorithm combined these features during its fitting process. It allowed for a more reliable use of measures in the face of sparse measurements. Such a method is widely used in ML of medical data^6,15–17^. We trained and tested the model on MIMIC-III data and on Rabin data, both with and without operational features. These operational features included *positive end-expiratory pressure set; tidal volume set; respiratory rate set; plateau pressure; positive end-expiratory pressure; tidal volume observed; fraction inspired oxygen set; tidal volume spontaneous; peak inspiratory pressure.* Extremely rarely measured features were excluded from consideration. This was inherent to the XGBoost method due to the decision tree structure, and rarely measured features are considered unreliable and are not considered when building the prediction model.

### Feature correlation matrix

The effect of spurious measurements and correlations was a concern. To address this issue, as well as to better understand the logics of the feature importance algorithm, we employed a correlation matrix that studied interactions between features and examined the correlation vector between features and outcomes. Correlations were aggregated and analyzed in two ways: once as a time driven “autoregressive” process and once as the pathophysiological influence of features on respiratory failure without their time dependencies.

Time driven analysis: this autoregressive analysis of data took all the features for each of the 6 h of the processing window with a further 6 h gap before the IMV outcome 4 h period. Static features were then added, and a Pearson correlation matrix was constructed to study the effect of features over time during the 6 h window. We then employed directed acyclic graph (DAG)^18^ to visually study the timeline dependencies. An example of this matrix and corresponding DAG are presented in Supplement 6 and the full correlation matrices are available at https://github.com/lshvartser1959/TSG-ICU.

Pathophysiological influence analysis: to analyze the pathophysiological meaning of XGBoost feature importance assignment, the time series were aggregated by hours and a second DAG was built to study the feature selection process. It follows the trajectories of the XGBoost importance assignment process. The feature chosen first is the feature with the highest importance. The correlations of each feature with the other features are drawn from the Pearson correlation matrix. The next features were chosen in a descending order of importance, a process that was performed repeatedly until the measured importance was lower than a chosen threshold. For each chosen feature, the features with which it had the best correlation were drawn. Features were then clustered in tiers, based on the Pearson correlation matrix. This process and its results for Rabin COVID 19 adapted model without operational features appear in Supplement 7.

### Ethics approval

The trial was approved by the institutional review board of the Rabin medical center. Consent was waived as this was an anonymized analysis of existing data.

## Results

The MIMIC-III non-COVID-19 cohort included 34,486 patients, of whom 13,328 (39%) required IMV at some stage, and 11,816 (34%) were hypoxemic. Of the hypoxemic patients, 6,281 (53%) received IMV at some stage. The Rabin cohort included 1,279 patients suffering from COVID-19, admitted between March 2020 and February 2021, of whom 163 required IMV during their hospitalization course. 1061 (83%) of the COVID-19 cohort were hypoxemic. 3 of the patients on IMV were not hypoxemic at any stage, i.e. ventilated for other reasons, leaving 160 patients receiving IMV for hypoxemic respiratory failure. Patient baseline characteristics are presented in Table 1.

**Table 1:** Baseline characteristics for hypoxemic patients only in the MIMIC III and the Rabin COVID-19 cohorts.

Characteristic	MIMIC III	Rabin medical center
Total number of hypoxemic patients	11,816	1061
Male sex	6589 (55.8%)	626 (59.0%)
Age- years	67.4 (± 16.2)	67.7 (± 16.5)
Weight- kg	82.7 (± 24.1)	79.3 (± 20.8)
Height- cm	168.8 (± 12.5)	166.5 (± 9.5)
BMI- kg/m^2^	29.8 (± 11.4)	28.7 (± 8.6)
**In the first 24 h**		
Heart rate- beats per minute	87.0 (± 17.9)	81.4 (± 16.7)
Mean blood pressure- mmHg	76.6 (± 14.8)	82.5 (± 17.5)
Platelets- 10^3^/microliter	204.7 (± 111.7)	218.9 (± 100.5)
Creatinine- mg/dl	1.5 (± 1.5)	1.4 (± 1.4)
Bilirubin- mg/dl	2.1 (± 4.5)	0.6 (± 1.6)
White Blood cells- 10^3^/microliter	13.3 (± 13.0)	7.4 (± 4.3)
PaO2/FiO_2_	297.2 (± 163.6)	148.8 (± 75.8)
SpO_2_/FiO_2_	187.7 (± 147.2)	170.7 (± 57.5)
**Respiratory support during stay**		
Oxygen therapy (other than high flow)	10,356 (87.6%)	308 (29.0%)*
High flow oxygen therapy	1031 (8.7%)	198 (18.7%)*
Non-invasive ventilation (CPAP or BiPAP)	854 (7.2%)	16 (1.6%)*
Invasive mechanical ventilation	6281 (53.2%)	160 (15.1%)*
**Outcome measures**		
Hospital length of stay- days	9.4 ($\pm 8.3)$	15.1 ($\pm 11.6)$
Hospital mortality	2289 (19.3%)	211 (20.0%)
Length of ventilation (only patients on IMV)- hours	41.1 ($\pm 46.3)$	176 ($\pm 230.7)$
SOFA	8.18 ($\pm 2.51)$	N/A

*BMI* body mass index, *CPAP* continuous positive airway pressure, *BiPAP* bilevel positive airway pressure, *IMV* invasive mechanical ventilation, *N/A* not available, *SOFA* sequential organ failure assessment.

*Recorded means of noninvasive respiratory support in Rabin are very low. This could only be attributed to underreporting of the modalities used.

Overall, there were 1,032,361 h of measurement in the MIMIC-III dataset and 254,905 h of measurement in the Rabin dataset. Statistics for the three different models, with and without operational features with statistics, are presented in Supplement 8.

**Figure 3:**
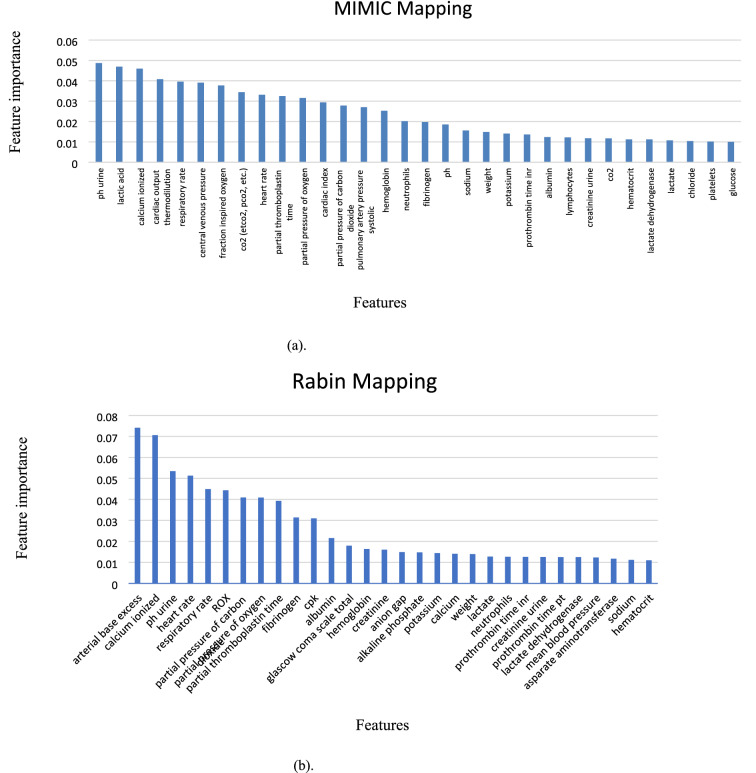
Feature importance in different feature mappings. a. MIMIC-III mapping, b. Rabin mapping. The relative contribution to the predictive model of the most important features for each database is shown. ROX index was defined as the ratio of pulse oximetry/fraction of inspired oxygen to respiratory rate^19^. Feature importance in Gradient Boosting Trees (XGBoost is one of them) is calculated for a single decision tree by the amount that each attribute split point improves the performance measure, weighted by the number of observations the node is responsible for. The feature importance is then averaged across all the decision trees within the model^20^.

The IMV onset prediction model developed and tested using the MIMIC-III (non-COVID-19) data yielded an area under the curve (AUC) of 0.91 when the operational features were included and an AUC of 0.83 when excluded. The AUC was 0.91 after 6 and 12 h and 0.90 after 18 and 24 h, meaning prediction quality degraded very little during the course of 24 h. The most important features for both MIMIC-III and Rabin datasets with an importance level above 0.01 are presented in Fig. 3 (a and b, respectively). XGBoost feature importance assignment takes care not to consider rarely measured features as important ones. Among the 25 most important features in MIMIC III, only the d-dimer had relatively few measurements (1572), but it was nonetheless included by the XGBoost model as this feature showed high importance in the prediction of deterioration. Rare measurement with less than 1,000 values are at the end of the list (Supplement 9). The feature importance for the 6-h gaps in MIMIC-III and Rabin configurations with operational features are presented in in Supplement 10.

We compared the feature importance ranking obtained from the XGBoost model with the ranking obtained from their correlations (absolute values) with IMV onset using the adapted models on the Rabin COVID-19 cohort, both with operative features and without them. There were 73 features in the analysis. They were divided according to their importance in the XGBoost model, separating the 36 more important features from the 37 less important ones. The same was done for the correlation sequence. The odds ratio without operational features was 3.20 and 2.97 with them. Considering the inaccuracy of both linear Pearson correlation and simple sorting by correlations, these odd ratios signify a positive association between the XGBoost sequence and the correlation sequence.

**Figure 4:**
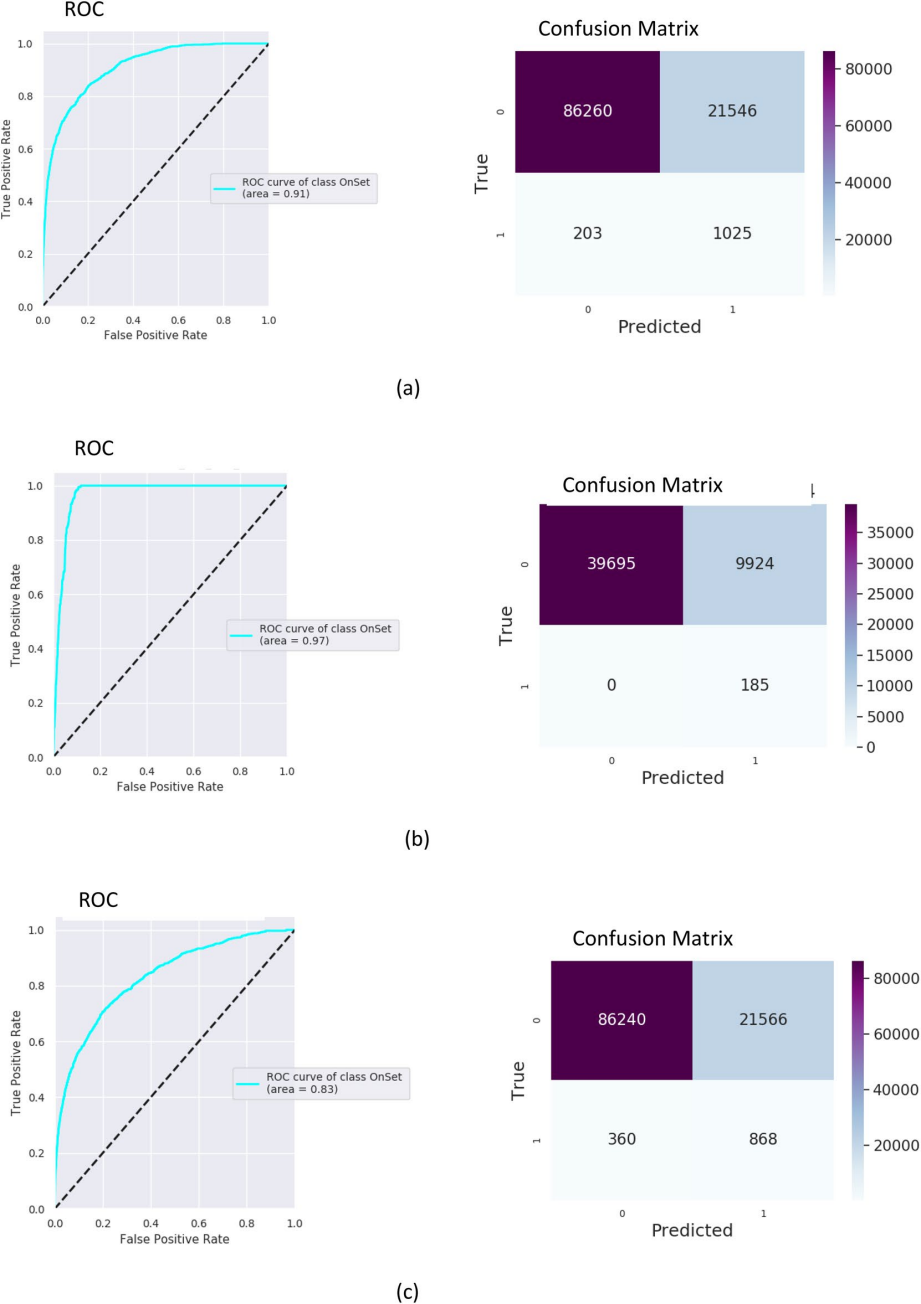

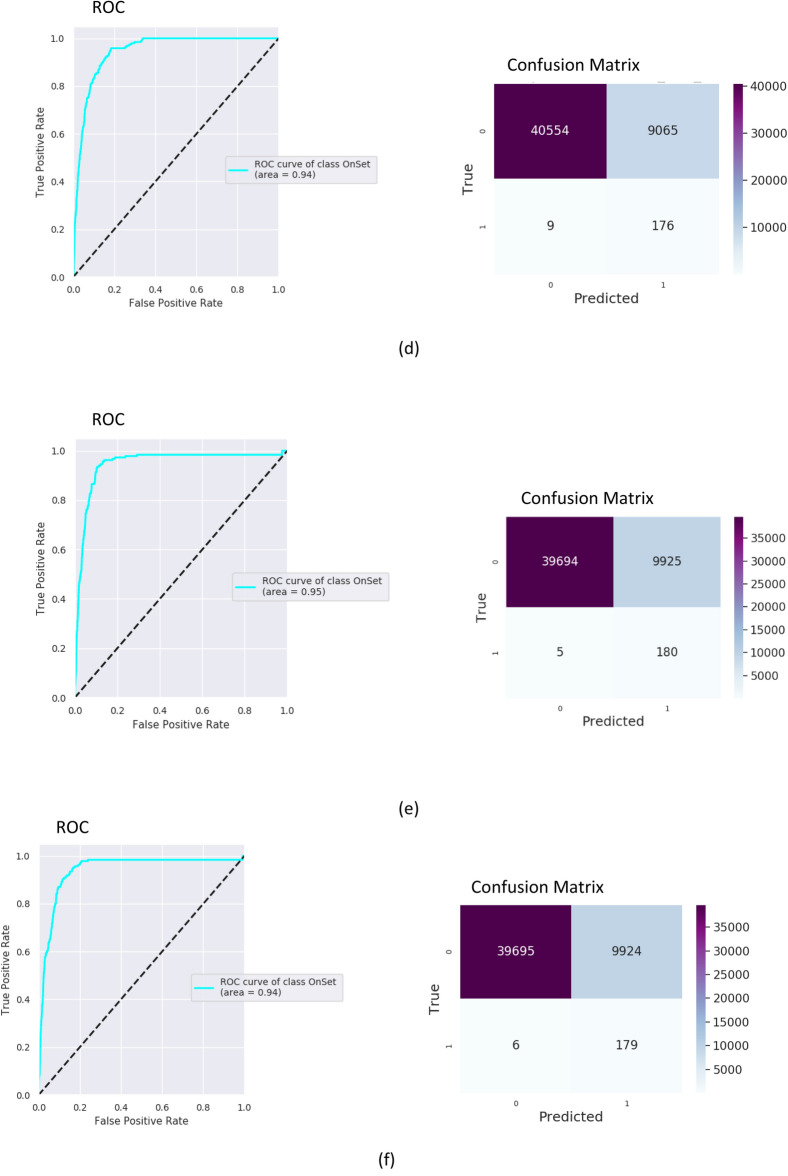
Testing receiver operator characteristic (ROC) curves and confusion matrices for MIMC III and the Rabin COVID-19 data using 6-h gaps for 1061 patients. An ROC curve is a graphical plot that illustrates the diagnostic ability of a binary classifier system as its discrimination threshold is varied. Confusion matrix summarizes the diagnostic ability of a binary classifier system when the threshold is chosen for the false positive rate of 0.2. Within the confusion matrix, cell (1,1) is the number of true positives, cell (0,0) is the number of true negatives, cell (0,1) is the number of false positives, cell (1,0) is the number of false negatives. Presented numbers are numbers of sliding window intervals used in testing procedure. (**a**) Training on MIMIC III with operational features. (**b**) Training on MIMIC-III with adaptation to Rabin COVID-19 and with operational features. (**c**) Training on MIMIC III without operational features. (**d**) Training on MIMIC-III with adaptation to Rabin COVID-19 and without operational features. (**e**) Training on Rabin COVID-19 with operational features. (**f**) Training on Rabin COVID-19 without operational features.

For assessment of the predictive abilities of the algorithm, a receiver operator characteristic (ROC) curve was created, with the area under the curve (AUC) calculated. Results of training and testing on MIMIC III with operational features are presented in Fig. 4. The model was trained on MIMIC-III (80% of MIMIC III hypoxemic data for training and 20% for testing), adapted on 70% of the Rabin data and tested on the remaining 30% of the Rabin data. Applying the MIMIC model “as is” (no adaptation) on the Rabin data of COVID-19 patients resulted with to AUC of 0.80 and 0.78 with and without the clinician’s settings respectively. After applying the adaptation on the COVID-19 data, the model yielded an AUC of 0.97 with the operational features and an AUC of 0.94 without them (Fig. 4). Results on Rabin data while not relying on MIMIC-III model achieved an AUC of 0.95 with the operational features and an AUC of 0.94 without them (Fig. 4). The confusion matrices were built with a false positive rate of ~ 0.2 for all experiments.

The algorithm runs once every hour, with a sliding time-window of measurements of 6 h. While maintaining a time-gap of 6 h, it predicts onset in a time-window of 4 h after the gap. Intervals with real onset in the measurements or gap windows don’t participate in training or testing. This method may lead to one true event to be predicted up to 4 times. Therefore, the number of events in the confusion matrix is the number of predictions and not the true number of patients, onsets or events.

**Figure 5:**
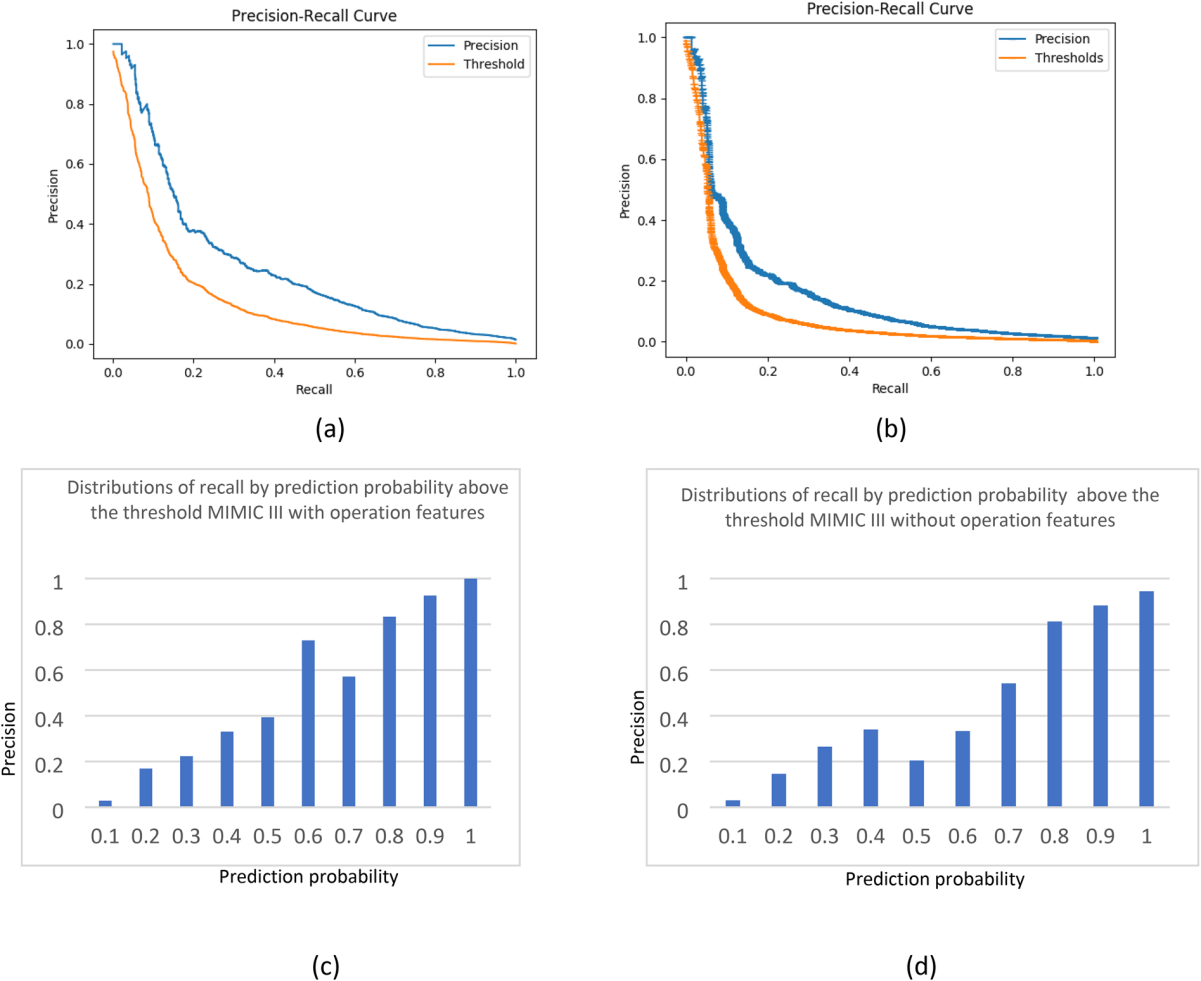
Precision-recall curves and distributions of precision by prediction probability above the threshold. (**a**,**c**) Training on MIMIC III with operational features. (**b**,**d**) Training on MIMIC III without operational features. Figures (**c**) and (**d**) were built as follows: prediction probability interval above the threshold was divided into the intervals [$\mu$, 0.1], [0.1, 0.2], … , [0.9, 1]. The chosen threshold ($\mu )$ for prediction probability corresponds with 20% of false positives. Precision was calculated for each interval separately.

The quality of recognition reflected by the AUC was high for all models. However, the false positive rate was also high. When analyzing the confusion matrices, F-scores and Matthew correlations (as shown in detail in Table 2), the precision (i.e., the number of true positives relative to the number of true positives and false positives) was poor for this threshold, even supposing that recall (i.e., true positive) was twice more important than precision. As the false positive rate was high, we analyzed the precision-recall curves and distributions of data by prediction probability. The precision-recall curves built for the MIMIC III cases are presented in Fig. 5. It is possible to increase the threshold, resulting in higher precision but recall is then decreased. In this scenario, patients that strongly require IMV will still be recognized, but borderline cases could potentially be missed. The chosen threshold ($\mu )$ for prediction probability corresponds with 20% of false positives. To calculate the precision, every interval after the threshold was analyzed accordingly: [$\mu$, 0.1], [0.1, 0.2], …, [0.9, 1]. Precision increased drastically as probability of prediction moved away from the threshold, most prominently when the probability was above 0.5.

**Table 2:** Different operational characteristics for computational experiments.

Data base	Model type	Operational features	AUC	F1-score,$\beta =0.5$	Matthews correlation coefficient
MIMIC III	Self model	Yes	0.91	0.19	0.17
MIMIC III	Self model	No	0.83	0.16	0.13
Rabin COVID-19 patients	Adaptation MIMIC—> Rabin	Yes	0.97	0.09	0.12
Rabin COVID-19 patients	Adaptation MIMIC—> Rabin	No	0.94	0.09	0.12
Rabin COVID-19 patients	Self model	Yes	0.95	0.08	0.12
Rabin COVID-19 patients	Self model	No	0.94	0.08	0.12

$\beta =0.5$—recall twice more important than precision. Matthew correlation coefficient (MCC) takes into account true and false positives and negatives and is generally regarded as a balanced measure which can be used even if the classes are of very different sizes. The MCC is in essence a correlation coefficient between the observed and predicted binary classifications; it returns a value between − 1 and + 1. A coefficient of + 1 represents a perfect prediction, 0 no better than random prediction and − 1 indicates total disagreement between prediction and observation. The ${\mathbf{F}}_{1}$ score is the harmonic mean of the precision and recall. The more generic ${\mathbf{F}}_{{\varvec{\upbeta}}}$ score applies additional weights, valuing one of precision or recall more than the other. The highest possible value of an F-score is 1.0, indicating perfect precision and recall, and the lowest possible value is 0, if either the precision or the recall is zero.

The number of positives in the Rabin dataset was small, making precision distributions and precision-recall curves meaningless, as there were many values around zero. Using an approximation process based on the similarity of distributions between the Rabin and MIMIC III testing sets, additional values were added to create precision distribution curves by probability. This process is discussed in detail in Supplement 11.

Sensitivity analysis was performed by splitting the sets of patients 30 times in random and calculating the 95% confidence intervals for AUC. The confidence intervals observed were rather tight, meaning the prediction level remained stable across different sets. These results are presented in Table 3.

**Table 3:** Confidence intervals for AUC in considered experiments.

Experiment	Confidence interval	
	Min	Max
MIMIC-III, with operational features	0.902	0.916
MIMIC-III, without operational features	0.822	0.837
Training, testing on Rabin COVID-19 with operational features	0.940	0.959
Training, testing on Rabin COVID-19 without operational features	0.931	0.947
Training on MIMIC-III with adaptation on Rabin COVID-19 and with operational features	0.961	0.975
Training on MIMIC-III with adaptation on Rabin COVID-19 and without operational features	0.932	0.946

AUC 95% confidence intervals were estimated by splitting the sets of patients 30 times in random. Confidence intervals were tight, meaning the prediction level remained stable across the different sets.

The feature correlation matrix was calculated as previously described (Fig. S1 in Suppl. 7). Features were clustered into tiers according to correlations. The first tier comprised mostly of arterial blood gas results. The second tier comprised mostly of respiratory and hemodynamic features. The third tier was the respiratory rate alone. The complete list of features according to tiers appears in Supplement 7 (Table. S1).

## Discussion

In the past decades we saw great advances in the field of respiratory care for acute respiratory failure, although the mortality rates and related morbidity are still high. Noninvasive methods are often used to compensate for the increased respiratory load, with generally good results. NIV may be safely used, and a recent meta-analysis^21^ showed reduced mortality using face masks and helmet NIV compared with standard oxygen therapy. High flow oxygen therapy was shown to decrease mortality and intubation rates in acute hypoxemic respiratory failure as well^22^. However, delay in IMV initiation may become a double-edged sword, not only for the aforementioned association with increased mortality. While ventilator-induced lung injury has long been recognized, recent years have seen an increasing body of knowledge concerning patient self-inflicted lung injury. Allowing the patient with a high potential for lung damage, for instance a high respiratory drive, to remain under noninvasive support may in fact translate later into worse outcomes when compared with earlier initiation of IMV^23^. COVID-19 patients requiring IMV face a very deadly disease, with mortality rates ranging from 35 to 97%^24^, well above those expected for general acute hypoxemic respiratory failure or even those with other viral pathogens.

Potential ways to discern those who are expected to fail under noninvasive support and for whom IMV might be better initiated earlier have been developed. Duan et al.^25^ developed a scale (HACOR) including heart rate, acidosis, consciousness, oxygenation, and respiratory rate to predict NIV failure. This method reached a diagnostic accuracy for NIV failure exceeding 80% in some subgroups. The application of this tool was also related with decreased hospital mortality rates, and its accuracy was later validated externally^26^. In another study using NIV after extubation^27^, the univariate analysis identified parameters as factors associated with reintubation. However, multivariate analysis identified pneumonia as the only predictive factor for failure of NIV-assisted extubation among critically ill patients.

Artificial intelligence and machine learning algorithms are increasingly employed to enhance prediction of respiratory deterioration and failure. Zeidberg et al.^28^ developed and studied two machine learning approaches, logistic regression and XGBoost, on patients with acute respiratory distress syndrome. L2 logistic regression performed best, reaching an area under the ROC curve of 0.81, identifying patients with four-fold greater chance to deteriorate. Several approaches have been studied specifically on COVID-19 populations. Ferrari et al.^29^ devised a hybrid approach combining machine learning tools, mainly ensemble decision trees, with the expertise of physicians, to predict 48 h in advance which patients would later develop moderately-severe respiratory failure, reaching 84% predictive accuracy. In the READY trial^30^ Burdick et al. employed XGBoost for fitting decision trees to detect patients who would deteriorate and require IMV after 24 h, reaching an AUC of 0.866. Most recently, an XGBoost-based model^31^ was employed to assess which COVID-19 patients would deteriorate after 48 h, reaching an AUC of 0.77, greatly outperforming an established early warning score.

Our model showed very high predictive accuracy for respiratory deterioration requiring invasive mechanical ventilation after time gaps of 6, 12, 18 and 24 h. Among the strongest predictive variables for MIMIC-III (non-COVID) patients were those related to and physiological parameters of cardio-respiratory function, including carbon dioxide (CO_2_) levels (both end-tidal and arterial partial pressure), actual respiratory rate and heart rate. Removal of the so-called operational features, led to a small degradation in the predictive quality of the models, but results were still robust. Validation of the method on COVID-19 data showed that the model could be used for the prediction of IMV onset for COVID-19 patients with high accuracy after the adaptations were integrated into our model. This promising tool, combining the two methods into a two-step model, may be of great aid in these times of COVID-19 Pandemic.

Our study suggests a model using an array of features, respiratory and non-respiratory, is able to predict respiratory failure at a very early stage. This detection may precede overt oxygenation or hemodynamic deterioration, potentially preventing emergency intubation and mechanical ventilation. These days this approach is potentially more relevant than ever, as the model's high accuracy was validated in COVID-19 patients. This two-step model could potentially be validated, adapted and trained in different settings and on various patient populations for early prediction of adverse outcomes.

The results showed that our model, trained on a specific dataset, could be adapted and used on a different dataset with different characteristics. Although the model was trained on a cohort of patients that were non-COVID-19, it could be adapted well to perform on a COVID-19 cohort at a different part of the world.

The AUCs for the models built were high, but this finding might be biased as the imbalanced sample of positive events/ negative events and it is reflected in the low F1 and Matthew scores. Threshold of false positives of 20% is only as an indication. To overcome this limitation and make the algorithm clinically applicable, a specific threshold that could be changed according to the clinical emphasis was set, allowing changes in the position on the precision-recall curve. Close to the threshold (probability lower than 0.2), precision was poor. In the mid ranges, between 0.2 and 0.8, the physician should be aware of an increasing risk for respiratory failure requiring IMV, with a higher risk the stronger the probability. With a probability above 0.8, IMV should be strongly considered. It is expected that clinicians using this tool will analyze the probabilities as trends in prediction, basing their decisions on repeated predictions and ongoing tendencies rather than solely on a single predictive probability, commonly known as a "snapshot", thus increasing the reliability of the model, and largely decreasing the false positive rate (i.e., increasing precision).

This research had several risks and limitations. A major risk was the inclusion of parameters that were largely irrelevant to the respiratory state of patients but showed spurious correlations. This was tackled using a correlation matrix and an examination of the XGBoost model feature importance ranking. We divided the features into tiers according to their ranking, i.e., relative contribution, to the prediction process. Even though data coverage in the Rabin cohort was sometimes low, our model used features according to their relative importance. Tiers were assigned to map the XGBoost algorithm, all of which are clusters of mutually correlated features. XGBoost chooses representative features from every cluster, a process that is explained in detail in Supplement 7. The algorithm found the most important features that were relevant for prediction, using alternate features from the same tier when the dedicated feature was sparse or missing.

There are currently very few existing investigations into combinations of feature selection and missing value imputation. Such an attempt was undertaken by Liu and colleagues in 2020^32^. One of the results of this work is that combination of the Decision Tree feature selection and imputation is a better choice for higher dimensional datasets. Despite investigating a simple model of missing values (the missing values occurred entirely at random, which means that the data were missing with no respect to both observed and unobserved data) and the fact that the results were evaluated only with the SVM classifier, these combinations of decision trees and imputation are the closest to our feature Importance scheme (XGBoost—gradient boosting tree) and imputation method (simple imputer). It is implicitly in support of our feature importance and imputation mechanisms.

Another important limitation of our work was poor data coverage for several features. Oxygen saturation, for example, was reported in 56,696 h of measurement (23%), despite being measured continuously for practically all patients. To overcome this hindrance, missing data for features were compensated by data on other features from the same tier. A future solution is the transition to systems that automatically store monitored data and allow better data reporting in the AI era.

A third limitation of our study was the need to connect different computerized information systems and event managers, creating a potential for registration and adaptation errors. A second limitation is our reliance in several decision trees, leading to only a moderate transparency of the model. This is partially offset, however, by the exact nature of feature importance, allowing to ascertain the relative part of each feature in the prediction process. Another limitation is the small size of the COVID-19 validation dataset. Further studies including larger populations may confirm our findings.

A fourth limitation was the imbalanced data that presented a serious potential problem. Undersampling of the major cases or oversampling of the minor cases over the training set is the standard way to overcome the problem of imbalanced data^32^. XGBoost contains an inner mechanism of weighting calculation of the stochastic gradient^33^, as mentioned in the feature importance section. An additional method to deal with imbalanced data in XGBoost without oversampling/undersampling, based on the same principal, was developed in 2020^34^, but it was not employed in our analysis. To make sure XGBoost solved the data imbalance in our case, we ran the XGBoost model without oversampling, with RandomOverSampler and SMOTE oversampling of imblearn Python package. The results are presented in Supplement 12. Comparing the values of AUC, sensitivity, and specificity for runs with and without oversampling, the numbers are very similar for all methods (Suppl. 12). XGBoost itself processed the unbalanced data with almost identical sensitivity and specificity compared with oversampling.

A fifth limitation was the lack of a unified definition of respiratory failure necessitating initiation of IMV. As acute respiratory failure and the need for intubation may in fact represent many different clinical phenotypes, differing according to subjective clinical assessments, this end point is potentially not an exact one. However, the need for intubation is an acceptable and often employed endpoint^22,35^. Moreover, to better select those who were intubated for respiratory failure, only hypoxemic patients were included in all analyses.

In its nature, this was a retrospective study, examining past data. Overt as well as unrecognized errors in data recording and translation may lead to significant bias, and only future analyzes on data from external sets as well as prospective evaluations may truly reveal the clinical utility and place in decision making of this newly devised tool.

## Conclusions

The prediction model adapted and trained by our team showed good predictive abilities for up to 24 h before initiation of invasive mechanical ventilation, potentially aiding in the decision to initiate invasive mechanical ventilation. The model has been trained and tested on a large patient cohort and proved highly accurate and may be superior to previously described models. However, low overall precision could still potentially limit clinical application, and the model's prediction should be interpreted according to probabilities and not as a yes/no question. It merits external validation by other teams to assess and further improve its performance on different settings and patient populations.

## Supplementary Information

Supplementary Information 1.

Supplementary Information 2.

Supplementary Information 3.

Supplementary Information 4.

Supplementary Information 5.

Supplementary Information 6.

Supplementary Information 7.

Supplementary Information 8.

Supplementary Information 9.

Supplementary Information 10.

Supplementary Information 11.

Supplementary Information 12.

## Data Availability

The code and the data to run this procedure and to evaluate it are available at https://github.com/lshvartser1959/TSG-ICU.

## Abbreviations

NIV: Non-invasive ventilation
IMV: Invasive mechanical ventilation
COVID-19: Corona virus disease 2019
MIMIC-III: Medical information mart for intensive care III
ML: Machine learning
ROC: Receiver operating characteristic
AUC: Area under the ROC curve
