# Supplementary material for: A novel machine learning model to predict respiratory failure and invasive mechanical ventilation in critically ill patients suffering from COVID-19
Source: Sci Rep. 2022 Jun 22;12:10573. doi: 10.1038/s41598-022-14758-x (PMC9216294; doi:10.1038/s41598-022-14758-x)
Supplement: Supplementary file 1 — Supplementary Information 1. [file 41598_2022_14758_MOESM1_ESM.docx]

**Supplement 1. Data unification and data matrix- implementation details**

The environment for the development of the Machine Learning algorithms was created in the cloud and contained the MIMIC III database, MIMIC Extract pipeline, Python, PyCharm developer studio and C++.

The data vector x+ for every sliding window is formed as concatenation of 6 values, value per hour for all the dynamic features plus static features (age, weight, etc.) added to the same vector. For example, if we had 100 dynamic measurements and 5 static measurements, our vector became of dimensions 100*6 + 5 = 605. The corresponding label y_t_ is 1 if the IMV Onset is detected in 4 hours after the gap and 0 otherwise. Every dynamic feature was presented with its mask, reflecting its reality and with time since last actual measurement, reflecting its accuracy. In feature importance calculation, all three importance types are summarized. This approach is accepted for the medical applications where the features are measured with different rates and imputer is used for data synchronization.

The hospital data for Rabin were transformed to the MIMIC-III tables in order to use the MIMIC Extract pipeline. The converter software loaded a set of text files (CSV) representing a snapshot of the medical database in Rabin medical center and converted them into a database in a format supported by MIMIC. The data items described patients' demographics, admissions, vital signs, laboratory results, treatments and more. The entire conversion software was developed in C++ as multi-platform and supports both Windows and Linux operating systems. The conversion software created the following tables: ADMISSIONS, ICUSTAYS, PATIENTS, CHARTEVENTS, LABEVENTS, D_ITEMS, D_LABITEMS.

The logic of labelling is to recognize in data the onset of intubation and the weaning time. These time stamps are recognized by rules formulated by the clinicians based on the manual records and device measurements. Weaning shall be defined in order not to miss possible reintubations. All the time interval between intubation and weaning is marked as IMV.
