# Supplementary material for: A novel machine learning model to predict respiratory failure and invasive mechanical ventilation in critically ill patients suffering from COVID-19
Source: Sci Rep. 2022 Jun 22;12:10573. doi: 10.1038/s41598-022-14758-x (PMC9216294; doi:10.1038/s41598-022-14758-x)
Supplement: Supplementary file 2 — Supplementary Information 2. [file 41598_2022_14758_MOESM2_ESM.docx]

**Supplement 2.** Rabin Mapping Features

| **Measurements & Calculated Features** | **Operational Features** |
| --- | --- |
| age | positive end-expiratory pressure set |
| alanine aminotransferase | tidal volume set |
| albumin | positive end-expiratory pressure |
| alkaline phosphate | peak inspiratory pressure |
| anion gap | fraction inspired oxygen |
| arterial base excess |  |
| asparate aminotransferase |  |
| basophils |  |
| bicarbonate |  |
| calcium |  |
| calcium ionized |  |
| chloride |  |
| chloride urine |  |
| cholesterol |  |
| cpk |  |
| creatinine |  |
| creatinine urine |  |
| crp |  |
| d-dimer |  |
| diastolic blood pressure |  |
| eosinophils |  |
| ferritin |  |
| fibrinogen |  |
| glascow coma scale total |  |
| glucose |  |
| heart rate |  |
| hematocrit |  |
| hemoglobin |  |
| lactate |  |
| lactate dehydrogenase |  |
| lymphocytes |  |
| lymphocytes atypical |  |
| magnesium |  |
| mean blood pressure |  |
| mean corpuscular hemoglobin |  |
| mean corpuscular hemoglobin concentration |  |
| mean_blood_pressure |  |
| neutrophils |  |
| oxygen saturation |  |
| partial pressure of carbon dioxide |  |
| partial pressure of oxygen |  |
| partial thromboplastin time |  |
| ph |  |
| ph urine |  |
| phosphorous |  |
| platelets |  |
| potassium |  |
| prothrombin time inr |  |
| prothrombin time pt |  |
| red blood cell count |  |
| respiratory rate |  |
| ROX |  |
| sodium |  |
| systolic blood pressure |  |
| temperature |  |
| total protein |  |
| total protein urine |  |
| troponin-t |  |
| weight |  |
| white blood cell count |  |
