# Supplementary material for: A novel machine learning model to predict respiratory failure and invasive mechanical ventilation in critically ill patients suffering from COVID-19
Source: Sci Rep. 2022 Jun 22;12:10573. doi: 10.1038/s41598-022-14758-x (PMC9216294; doi:10.1038/s41598-022-14758-x)
Supplement: Supplementary file 3 — Supplementary Information 3. [file 41598_2022_14758_MOESM3_ESM.docx]

**Supplement 3.** The adaptation algorithm

The adaptation algorithm works as follows:

1. Train model on source data - M_S_ (MIMIC-III). Save it as “ms.model”.
2. Take a part t_α_ of imbalanced target patients (Rabin) for adaptation and another part v_α_ of imbalanced target patient data for validation.
3. Take source model M_S_ and increment its training on t_α_. Incremental training is done in such a way that the trees of M_s_ are working as is and the new trees are built only on the residuals (use **M_s_.fit(t_α_, y_tα_, xgb_model =’ms.model’)**). Obtain model M_T_.
4. Check M_T_ on the validation set (v_α_, y_vα_). Obtain AUC.
5. Repeat 3-5 with different small parts of data and choose a model with M_T_^*^ with best AUC.

Remove the small retraining and validation parts of data, corresponding to MT* from the target data and check the AUC on the remained part.
