# Supplementary material for: A novel machine learning model to predict respiratory failure and invasive mechanical ventilation in critically ill patients suffering from COVID-19
Source: Sci Rep. 2022 Jun 22;12:10573. doi: 10.1038/s41598-022-14758-x (PMC9216294; doi:10.1038/s41598-022-14758-x)
Supplement: Supplementary file 4 — Supplementary Information 4. [file 41598_2022_14758_MOESM4_ESM.docx]

**Supplement 4.** Evaluation of the adaptation algorithm

Initial evaluation of adaptation algorithm was performed on a dataset of 3,000 admissions from MIMIC III, in which 311 of them were detected as non-ARDS and 2689 were with ARDS. Presence of ARDS was detected when PEEP set > 5 cmH2O and PaO2/FiO2 ratio < 300 mm Hg. Patients with heart failure were excluded from the ARDS cohort. Heart failure patients were excluded because they are treated with the same simplified conditions PEEP set > 5 cmH2O and PaO2/FiO2 ratio < 300 mm Hg both when they have ARDS and when they don’t. The Source Set was the non-ARDS and the Target Set was with ARDS. The IMV Onset prediction was trained and tested according to the scheme described above for 6 hours gap. The results obtained are presented (Table 2), p is a part of target data taken for validation and for testing.

Adaptation algorithm initial evaluation on MIMIC non-ARDS/with ARDS. After the adaptation update on 0% - 50% of ARDS data, the prediction strength increased in 15% from AUC = 0.62 to AUC = 0.77. p is a part of target data taken for validation and for testing.

| Training | Validation/Testing | AUC |
| --- | --- | --- |
| Source Set | Testing: Target Set | 0.62 |
| Source Set + p=0.1 | Validation: p=0.1 | 0.6 |
| Source Set + p=0.2 | Validation: p=0.2 | 0.7 |
| Source Set + p=0.3 | Validation: p=0.3 | 0.71 |
| Source Set + p=0.4 | Validation: p=0.4 | 0.72 |
| Source Set + p=0.5 | Validation: p=0.5 | 0.77 |
| Source Set + p=0.5 | Testing: 1-(p*(1+p))=0.25 | 0.77 |
