# Supplementary material for: A novel machine learning model to predict respiratory failure and invasive mechanical ventilation in critically ill patients suffering from COVID-19
Source: Sci Rep. 2022 Jun 22;12:10573. doi: 10.1038/s41598-022-14758-x (PMC9216294; doi:10.1038/s41598-022-14758-x)
Supplement: Supplementary file 5 — Supplementary Information 5. [file 41598_2022_14758_MOESM5_ESM.docx]

Options of training/testing/adaptation

Options of training/testing/adaptation executed on MIMIC III/ Rabin. On MIMIC data we ran training and testing both with and without operational features, i.e., features derived from equipment settings, defined by medical staff. On the Rabin data the scheme with and without operational features with and without adaptations were tested.

|  | **Self/Self** | | | | MIMIC/Hospital w/o adaptation | | | | MIMIC/Hospital with adaptation | | | |
| --- | --- | --- | --- | --- | --- | --- | --- | --- | --- | --- | --- | --- |
|  | **MIMIC map** | | Hospital map | | MIMIC map | | Hospital map | | MIMIC map | | Hospital map | |
|  | **with operative** | w/o operative | with operative | w/o operative | with operative | w/o operative | with operative | w/o operative | with operative | w/o operative | with operative | w/o operative |
| MIMIC | V | V | V | V |  |  |  |  |  |  |  |  |
| Rabin |  |  | V | V |  |  | V | V |  |  | V | V |
