# Supplementary material for: A novel machine learning model to predict respiratory failure and invasive mechanical ventilation in critically ill patients suffering from COVID-19
Source: Sci Rep. 2022 Jun 22;12:10573. doi: 10.1038/s41598-022-14758-x (PMC9216294; doi:10.1038/s41598-022-14758-x)
Supplement: Supplementary file 6 — Supplementary Information 6. [file 41598_2022_14758_MOESM6_ESM.docx]

**Supplement 6**. Causal dependencies in the “autoregressive” process of IMV onset prediction

The scheme used in the prediction model can be presented with help of a directed acyclic graph (DAG) presented on Figure S1. Every feature is taken during the 6 hours of measurements. Features are taken with their levels of confidence, reflected by their masks and time since last measurement. Every feature measured during a previous hour causally influences itself measured current hour and the possibility of IMV outcome. For simplicity only a single feature dependencies are presented on the graph.

In order to evaluate significances of all these dependencies we calculated the Pearson correlation coefficients between all the features participated in the prediction model (cross-correlations) and correlations between the features and the outcome. For every feature, correlations from its 3 sub-features maximal correlation were kept. Full correlation matrices are available at <https://github.com/lshvartser1959/TSG-ICU>. The submatrices for 5 most correlated features presented on Figures S2, S3. Correlations between the five significant features and outcome presented on Figure S2. Hours in measurement window are measured in opposite time: “_x” means hour (x-1) from the end of the measurement window. “_1” means the last hour in the measurement window, “_2” means the last hour in the measurement window and so on. Analyzing these results one can see that the correlations are small because the binary (“0”,”1”) scale of the outcome. It means that the classical autoregressive methods of significancy analysis are unapplicable. From the other side one can see that the correlation of every hour of feature is influences the outcome with approximately same strength but the strengths are slightly different from hour to hour. Figure S3 presents the cross correlations and correlations with outcome of the five significant features. As redder is color as higher the correlation. Figure S4 presents” zoom” for cross correlations and correlations with outcome for only one feature. As redder is color as higher the correlation. One can see that despite the high cross correlation between the measurements of the same feature in different hours the correlations are less than one and decreased with time. This “autoregressive” analysis prompts us that every one of 6 hours measurements has its impact to the outcome.

**Figure S1.** DAG of causal dependencies in “autoregressive” process of IMV onset prediction

Hour 6

Hour 5

Hour 4

Hour 3

Hour 1

Hour 2

6 more hours GAP

**Figure S2.** Correlations between the five significant features and outcome. Hours in measurement window are measured in opposite time: “_x” means hour (x-1) from the end of the measurement window. “_1” means the last hour in the measurement window, “_2” means the last hour in the measurement window and so on.

| **feature** | **vent** |
| --- | --- |
| ROX_1 | 0.189 |
| ROX_2 | 0.188 |
| ROX_3 | 0.187 |
| ROX_4 | 0.187 |
| ROX_5 | 0.188 |
| ROX_6 | 0.188 |
| arterial base excess_1 | 0.104 |
| arterial base excess_2 | 0.104 |
| arterial base excess_3 | 0.104 |
| arterial base excess_4 | 0.105 |
| arterial base excess_5 | 0.105 |
| arterial base excess_6 | 0.105 |
| calcium ionized_1 | 0.148 |
| calcium ionized_2 | 0.154 |
| calcium ionized_3 | 0.158 |
| calcium ionized_4 | 0.160 |
| calcium ionized_5 | 0.160 |
| calcium ionized_6 | 0.161 |
| fraction inspired oxygen_1 | 0.177 |
| fraction inspired oxygen_2 | 0.175 |
| fraction inspired oxygen_3 | 0.177 |
| fraction inspired oxygen_4 | 0.179 |
| fraction inspired oxygen_5 | 0.180 |
| fraction inspired oxygen_6 | 0.183 |
| partial pressure of oxygen_1 | 0.214 |
| partial pressure of oxygen_2 | 0.215 |
| partial pressure of oxygen_3 | 0.215 |
| partial pressure of oxygen_4 | 0.216 |
| partial pressure of oxygen_5 | 0.217 |
| partial pressure of oxygen_6 | 0.217 |

**Figure S3.** Cross correlations and correlations with outcome of the five significant features. As redder is color as higher the correlation.

Figure S4. ”Zoom” for cross correlations and correlations with outcome for only one feature. . As redder is color as higher the correlation.

|  | ROX_1 | ROX_2 | ROX_3 | ROX_4 | ROX_5 | ROX_6 | vent |
| --- | --- | --- | --- | --- | --- | --- | --- |
| ROX_1 | 1.000 | 0.988 | 0.977 | 0.967 | 0.958 | 0.949 | 0.189 |
| ROX_2 | 0.988 | 1.000 | 0.988 | 0.977 | 0.966 | 0.957 | 0.188 |
| ROX_3 | 0.977 | 0.988 | 1.000 | 0.987 | 0.976 | 0.965 | 0.187 |
| ROX_4 | 0.967 | 0.977 | 0.987 | 1.000 | 0.987 | 0.976 | 0.187 |
| ROX_5 | 0.958 | 0.966 | 0.976 | 0.987 | 1.000 | 0.987 | 0.188 |
| ROX_6 | 0.949 | 0.957 | 0.965 | 0.976 | 0.987 | 1.000 | 0.188 |
