# Supplementary material for: A novel machine learning model to predict respiratory failure and invasive mechanical ventilation in critically ill patients suffering from COVID-19
Source: Sci Rep. 2022 Jun 22;12:10573. doi: 10.1038/s41598-022-14758-x (PMC9216294; doi:10.1038/s41598-022-14758-x)
Supplement: Supplementary file 7 — Supplementary Information 7. [file 41598_2022_14758_MOESM7_ESM.docx]

**Supplement 7:** The clustering process

We set to show that the XGBoost feature importance assignment process chooses features according to their clinical (pathophysiological) importance. In order to do so, a DAG that is following the trajectories of the XGBoost importance assignment process was built. The feature chosen first is the feature with the highest importance. The correlations of each feature with the other features are drawn from the Pearson correlation matrix. The next features were chosen in a descending order of importance, a process that was performed repeatedly until the measured importance was lower than a chosen threshold (we choose the threshold 15% of maximal score value, because the features with score below this threshold seemed much less important pathophysiologically). For each chosen feature, the features with which it had the best correlation were drawn. Features were than clustered in tiers based on the Pearson correlation matrix.

This directed acyclic graph (DAG) of the feature correlation matrix is presented oh Figure S1. Features are examined repeatedly, starting with the most important feature. The blue arrows illustrate the Pearson correlations between the features greater than 0.6, moving from the most important feature to the features best correlated with it. The red arrows follow the trajectory of the XGBoost feature importance process, moving from the highest importance downwards in order of importance. A solid red arrow represents the features that follow in order of importance and are also correlated. A dashed red arrow represents the features that follow in order of importance but are not correlated.

Features were clustered into tiers according to correlations. We used a simple algorithm following the DAG and cut off the current cluster when the number of arks connected to the following nodes is less than a percent (we used one third) of its inner arcs. The complete list of features according to tiers appears in Table S1.

The first and most influential tier mainly comprised of results of arterial blood gas analysis, while the second and third tier mainly contained vital signs with an emphasis on respiratory parameters, all of which are clinically crucial when deciding on IMV initiation. Other tiers included many other parameters with varying clinical relation to respiratory failure, but this process largely avoided the risk of spurious correlations, as features of lower clinical relevance were mostly located in the lower tiers.

An example of a part of the most influenced features among themselves and with IMV onset sorted by absolute value of correlation with onset presented in Table S2. The full matrix of correlations can be seen <https://github.com/lshvartser1959/TSG-ICU>. Feature importances aggregated by hours and by subfeatures (max operator). XGBoost feature importances also aggregate by hours and by subfeatures (sum operator).

**Figure S1.** A directed acyclic graph (DAG) of the feature correlation matrix. Features are examined repeatedly, starting with the most important feature. The blue arrows illustrate the Pearson correlations between the features greater than 0.6, moving from the most important feature to the features best correlated with it. The red arrows follow the trajectory of the XGBoost feature importance process, moving from the highest importance downwards in order of importance. A solid red arrow represents features that follow in order of importance and are also correlated. A dashed red arrow represents features that follow in order of importance but are not correlated.

| Tier number | Features |
| --- | --- |
| 1 | arterial base excess, calcium ionized, partial pressure of oxygen, anion gap, chloride, hemoglobin, bicarbonate, lactate, ph, partial pressure of carbon dioxide, potassium, sodium |
| 2 | ROX, heart rate, systolic blood pressure, oxygen saturation, fraction inspired oxygen, mean blood pressure, glasgow coma scale total, temperature, diastolic blood pressure |
| 3 | respiratory rate |
| 4 | Fibrinogen, d-dimer, partial thromboplastin time, prothrombin time inr, prothrombin time pt |
| 5 | weight, height |
| 6 | cpk, troponin-t, alkaline phosphate, lactate dehydrogenase, phosphorous, ferritin, bilirubin, crp, alanine aminotransferase, cholesterol hdl, cholesterol, albumin, total protein, calcium |
| 7 | ph urine, total protein urine |
| 8 | white blood cell count, eosinophils, neutrophils, mean corpuscular volume, creatinine, asparate aminotransferase, lymphocytes, monocytes, lymphocytes percent, mean corpuscular hemoglobin,  mean corpuscular hemoglobin concentration, red blood cell count, platelets, glucose, basophils, magnesium |
| 9 | hematocrit |

**Table S1.** The complete list of features in each tier.

|  | abs_vent | vent | partial pressure of oxygen | ROX | fraction inspired oxygen | white blood cell count | troponin-t | ph urine | hemoglobin | respiratory rate | bicarbonate |
| --- | --- | --- | --- | --- | --- | --- | --- | --- | --- | --- | --- |
| abs_vent | 1.00 | 1.00 | 0.22 | 0.19 | 0.18 | 0.18 | 0.17 | 0.16 | 0.16 | 0.16 | 0.16 |
| vent | 1.00 | 1.00 | 0.22 | 0.19 | 0.18 | 0.18 | 0.17 | 0.16 | 0.16 | 0.16 | 0.16 |
| partial pressure of oxygen | 0.22 | 0.22 | 1.00 | 0.36 | 0.11 | 0.48 | 0.34 | 0.07 | 0.98 | 0.13 | 0.99 |
| ROX | 0.19 | 0.19 | 0.36 | 1.00 | 0.19 | 0.10 | 0.13 | 0.19 | 0.36 | 0.43 | 0.37 |
| fraction inspired oxygen | 0.18 | 0.18 | 0.11 | 0.19 | 1.00 | 0.11 | 0.07 | 0.03 | 0.14 | 0.36 | 0.11 |
| white blood cell count | 0.18 | 0.18 | 0.48 | 0.10 | 0.11 | 1.00 | 0.31 | 0.11 | 0.49 | 0.16 | 0.49 |
| troponin-t | 0.17 | 0.17 | 0.34 | 0.13 | 0.07 | 0.31 | 1.00 | 0.14 | 0.34 | 0.07 | 0.34 |
| ph urine | 0.16 | 0.16 | 0.07 | 0.19 | 0.03 | 0.11 | 0.14 | 1.00 | 0.06 | 0.10 | 0.15 |
| hemoglobin | 0.16 | 0.16 | 0.98 | 0.36 | 0.14 | 0.49 | 0.34 | 0.06 | 1.00 | 0.14 | 1.00 |
| respiratory rate | 0.16 | 0.16 | 0.13 | 0.43 | 0.36 | 0.16 | 0.07 | 0.10 | 0.14 | 1.00 | 0.14 |
| bicarbonate | 0.16 | 0.16 | 0.99 | 0.37 | 0.11 | 0.49 | 0.34 | 0.15 | 1.00 | 0.14 | 1.00 |

**Table S2**. An example of a part of the most influenced features among themselves and with IMV onset sorted by absolute value of correlation with onset (abs_vent).
