# Supplementary material for: A novel machine learning model to predict respiratory failure and invasive mechanical ventilation in critically ill patients suffering from COVID-19
Source: Sci Rep. 2022 Jun 22;12:10573. doi: 10.1038/s41598-022-14758-x (PMC9216294; doi:10.1038/s41598-022-14758-x)
Supplement: Supplementary file 8 — Supplementary Information 8. [file 41598_2022_14758_MOESM8_ESM.docx]

**Supplement 8.** Models built and examined only for critically ill patients suffering from hypoxemia.

| Data Base | Model Type | Operational features | Overall number of patients | Number of hypoxemic respiratory failure critically ill patients | Number of hypoxemic respiratory failure critically ill IMV patients | Overall number of hours of measurements for hypoxemic respiratory failure critically ill patients |
| --- | --- | --- | --- | --- | --- | --- |
| MIMIC III | Self model | yes | 34486 | 11816 | 6281 | 1032361 |
| MIMIC III | Self model | no | 34486 | 11816 | 6281 | 1032361 |
| Rabin Medical Center COVID-19 | Adaptation MIMIC -> Rabin | yes | 1279 | 1061 | 160 | 254905 |
| Rabin Medical Center COVID-19 | Adaptation MIMIC -> Rabin | no | 1279 | 1061 | 160 | 254905 |
| Rabin Medical Center COVID-19 | Self model | yes | 1279 | 1061 | 160 | 254905 |
| Rabin Medical Center COVID-19 | Self model | no | 1279 | 1061 | 160 | 254905 |

Self model– trained and tested on a same database. Adaptation – adapted to Rabin COVID patients with our two-step solution.

About one third of MIMIC III patients were hypoxemic, of whom over 50% received IMV at some stage. Among the COVID-19 patients in Rabin medical center, 83% were hypoxemic, of whom 15% received IMV.

IMV- invasive mechanical ventilation.
