# Supplementary material for: A novel machine learning model to predict respiratory failure and invasive mechanical ventilation in critically ill patients suffering from COVID-19
Source: Sci Rep. 2022 Jun 22;12:10573. doi: 10.1038/s41598-022-14758-x (PMC9216294; doi:10.1038/s41598-022-14758-x)
Supplement: Supplementary file 9 — Supplementary Information 9. [file 41598_2022_14758_MOESM9_ESM.docx]

**Supplement 9.** Counters of times each feature to be observable in MIMIC III and in Rabin COVID data

Counters of times each feature to be observable in MIMIC III and in Rabin COVID data.

Real number of measurements presented in the column “counts”. Algorithm input is the averages per hour. This number of hours of measurements presented in the column “number of hours”. Overall number of hours of measurements presented in the Supplement 8.

The features presented in order of the importance assignment that XGBoost made. The features with score 0 were removed. It is seen that the poor measured features do not appear among the most important ones.

| Importance | Feature name | Number of measurements | Number of hours |
| --- | --- | --- | --- |
| 1 | arterial base excess | 89944 | 84917 |
| 2 | calcium ionized | 108968 | 62846 |
| 3 | partial pressure of oxygen | 59994 | 57890 |
| 4 | ROX | 62186 | 56645 |
| 5 | fraction inspired oxygen | 62186 | 56645 |
| 6 | hemoglobin | 159334 | 96948 |
| 7 | ph urine | 9910 | 9834 |
| 8 | partial pressure of carbon dioxide | 199618 | 108915 |
| 9 | cpk | 15805 | 15583 |
| 10 | respiratory rate | 1296457 | 923976 |
| 11 | fibrinogen | 19686 | 10219 |
| 12 | partial thromboplastin time | 125309 | 63561 |
| 13 | weight | 37774 | 32472 |
| 14 | ph | 277008 | 119438 |
| 15 | d-dimer | 1572 | 790 |
| 16 | albumin | 23941 | 12784 |
| 17 | potassium | 263850 | 117302 |
| 18 | bilirubin | 39580 | 20425 |
| 19 | heart rate | 1152421 | 938835 |
| 20 | lactate dehydrogenase | 9490 | 9444 |
| 21 | glascow coma scale total | 173376 | 172743 |
| 22 | phosphorous | 79268 | 54018 |
| 23 | monocytes | 11162 | 10936 |
| 24 | neutrophils | 11456 | 11220 |
| 25 | temperature | 624728 | 323557 |
| 26 | lactate | 39568 | 35065 |
| 27 | cholesterol ldl | 938 | 935 |
| 28 | calcium | 132982 | 70268 |
| 29 | basophils | 7141 | 7032 |
| 30 | magnesium | 165046 | 85439 |
| 31 | troponin-t | 9625 | 9478 |
| 32 | prothrombin time inr | 117661 | 59788 |
| 33 | prothrombin time pt | 117643 | 59779 |
| 34 | oxygen saturation | 1201804 | 903891 |
| 35 | creatinine | 169788 | 89568 |
| 36 | alanine aminotransferase | 30102 | 19963 |
| 37 | mean corpuscular hemoglobin | 65333 | 63927 |
| 38 | glucose | 440227 | 256245 |
| 39 | hematocrit | 203398 | 115768 |
| 40 | eosinophils | 187 | 184 |
| 41 | lymphocytes | 11378 | 11142 |
| 42 | bicarbonate | 100208 | 88214 |
| 43 | asparate aminotransferase | 30056 | 19933 |
| 44 | red blood cell count | 100558 | 64003 |
| 45 | sodium | 202797 | 103158 |
| 46 | height | 5880 | 2943 |
| 47 | white blood cell count | 189159 | 80707 |
| 48 | creatinine urine | 3395 | 3388 |
| 49 | platelets | 134542 | 84355 |
| 50 | total protein urine | 371 | 371 |
| 51 | mean corpuscular hemoglobin concentration | 65399 | 63993 |
| 52 | cholesterol hdl | 1004 | 1001 |
| 53 | chloride urine | 1297 | 1295 |
| 54 | anion gap | 94852 | 83865 |
| 55 | alkaline phosphate | 29276 | 19441 |
| 56 | mean corpuscular volume | 65334 | 63928 |
| 57 | lymphocytes percent | 58 | 58 |
| 58 | creatinine body fluid | 20 | 20 |
| 59 | crp | 110 | 110 |
| 60 | mean blood pressure | 1214969 | 909064 |
| 61 | ferritin | 1040 | 1040 |
| 62 | cholesterol | 2337 | 1202 |
| 63 | lymphocytes atypical | 1070 | 1065 |
| 64 | systolic blood pressure | 1205491 | 912819 |
| 65 | total protein | 408 | 225 |
| 66 | chloride | 183511 | 97519 |

**Table S1.** MIMIC III features without the operational ones in order to their importance with counted number of times their being observed. The features with score 0 were removed. The input to the algorithm included measurements averaged per hour for any that included at least one measurement.

| Importance | Feature name | Number of measurements | Number of hours |
| --- | --- | --- | --- |
| 1 | arterial base excess | 1267 | 1258 |
| 2 | calcium ionized | 11599 | 11223 |
| 3 | partial pressure of oxygen | 11538 | 11154 |
| 4 | ROX | 25010 | 9188 |
| 5 | fraction inspired oxygen | 29030 | 9188 |
| 6 | hemoglobin | 11278 | 10915 |
| 7 | partial pressure of carbon dioxide | 11539 | 11161 |
| 8 | respiratory rate | 66466 | 20960 |
| 9 | fibrinogen | 4656 | 4591 |
| 10 | cpk | 3004 | 2993 |
| 11 | ph urine | 685 | 678 |
| 12 | partial thromboplastin time | 4647 | 4588 |
| 13 | weight | 773 | 577 |
| 14 | potassium | 19955 | 16579 |
| 15 | d-dimer | 2893 | 2865 |
| 16 | ph | 11668 | 11284 |
| 17 | bilirubin | 8003 | 7942 |
| 18 | heart rate | 92132 | 43415 |
| 19 | glascow coma scale total | 2118 | 2095 |
| 20 | phosphorous | 8056 | 7994 |
| 21 | albumin | 8076 | 8015 |
| 22 | lactate | 11479 | 11116 |
| 23 | neutrophils | 8506 | 8408 |
| 24 | troponin-t | 3103 | 3092 |
| 25 | cholesterol ldl | 501 | 499 |
| 26 | oxygen saturation | 111134 | 56696 |
| 27 | magnesium | 3717 | 3672 |
| 28 | lactate dehydrogenase | 7562 | 7504 |
| 29 | prothrombin time pt | 4648 | 4591 |
| 30 | glucose | 20713 | 20118 |
| 31 | calcium | 8072 | 8010 |
| 32 | prothrombin time inr | 4647 | 4591 |
| 33 | alanine aminotransferase | 7822 | 7760 |
| 34 | monocytes | 8503 | 8405 |
| 35 | temperature | 67540 | 36272 |
| 36 | crp | 6995 | 6950 |
| 37 | basophils | 8010 | 7927 |
| 38 | mean corpuscular hemoglobin | 8512 | 8416 |
| 39 | asparate aminotransferase | 7486 | 7427 |
| 40 | creatinine | 8843 | 8765 |
| 41 | mean blood pressure | 40934 | 39502 |
| 42 | sodium | 20191 | 16684 |
| 43 | hematocrit | 8574 | 8476 |
| 44 | eosinophils | 6573 | 6503 |
| 45 | platelets | 8518 | 8419 |
| 46 | red blood cell count | 8515 | 8418 |
| 47 | bicarbonate | 11420 | 11053 |
| 48 | height | 391 | 390 |
| 49 | lymphocytes atypical | 159 | 159 |
| 50 | creatinine urine | 52 | 49 |
| 51 | lymphocytes percent | 8509 | 8411 |
| 52 | white blood cell count | 8516 | 8418 |
| 53 | diastolic blood pressure | 70596 | 28564 |
| 54 | mean corpuscular hemoglobin concentration | 8512 | 8416 |
| 55 | lymphocytes | 8485 | 8395 |
| 56 | ferritin | 2698 | 2665 |
| 57 | total protein urine | 577 | 569 |
| 58 | systolic blood pressure | 92923 | 39481 |
| 59 | chloride urine | 106 | 103 |
| 60 | cholesterol hdl | 2272 | 2262 |
| 61 | mean corpuscular volume | 8515 | 8418 |
| 62 | alkaline phosphate | 7930 | 7869 |
| 63 | cholesterol | 2278 | 2268 |
| 64 | chloride | 12643 | 11505 |
| 65 | anion gap | 3382 | 3354 |
| 66 | creatinine body fluid | 2 | 2 |
| 67 | total protein | 8068 | 8005 |

**Table S2.** Rabin COVID-19 features without the operational ones in order to their importance with counted number of times their being observed. The features with score 0 were removed. The input to the algorithm included measurements averaged per hour for any that included at least one measurement.
