# Supplementary material for: A novel machine learning model to predict respiratory failure and invasive mechanical ventilation in critically ill patients suffering from COVID-19
Source: Sci Rep. 2022 Jun 22;12:10573. doi: 10.1038/s41598-022-14758-x (PMC9216294; doi:10.1038/s41598-022-14758-x)
Supplement: Supplementary file 10 — Supplementary Information 10. [file 41598_2022_14758_MOESM10_ESM.docx]

**Supplement 10.** The 20 Most important features for the models including the operational features, i.e., those set by the clinician, namely ventilator settings.

| **Most influenced features including the operational features** | |
| --- | --- |
| **MIMIC mapping** | **Rabin mapping** |
| positive end-expiratory pressure set | positive end-expiratory pressure set |
| tidal volume set | mean airway pressure |
| respiratory rate set | ph urine |
| ph urine | tidal volume set |
| heart rate | ROX |
| partial thromboplastin time | positive end-expiratory pressure |
| fraction inspired oxygen set | heart rate |
| positive end-expiratory pressure | partial thromboplastin time |
| co2 (etco2, pco2, etc.) | hemoglobin |
| hemoglobin | peak inspiratory pressure |
| fraction inspired oxygen | calcium ionized |
| respiratory rate | fraction inspired oxygen |
| tidal volume spontaneous | partial pressure of carbon dioxide |
| albumin | albumin |
| weight | respiratory rate |
| peak inspiratory pressure | partial pressure of oxygen |
| plateau pressure | anion gap |
| glasgow coma scale total | cpk |
| central venous pressure | lactate |
| oxygen saturation | peak inspiratory pressure |
