# Supplementary material for: A novel machine learning model to predict respiratory failure and invasive mechanical ventilation in critically ill patients suffering from COVID-19
Source: Sci Rep. 2022 Jun 22;12:10573. doi: 10.1038/s41598-022-14758-x (PMC9216294; doi:10.1038/s41598-022-14758-x)
Supplement: Supplementary file 11 — Supplementary Information 11. [file 41598_2022_14758_MOESM11_ESM.docx]

**Supplement 11.** The creation of precision distribution curves by probability.

The number of positives in the Rabin dataset was small, making precision distributions and precision-recall curves meaningless, as there were many values around zero (Table S1). In order to allow any analysis of precision when the prediction probability was high (left part of the precision-recall curve), positives could be added to the training sample by approximation. While this method was not accurate, it still allowed phenomenological sensing.

The Rabin and MIMIC III testing sets showed very similar distributions of positive points, with correlation coefficients between 0.989 and 0.998 both with and without operational features, implying high similarity, as shown in table S2. Linear or quadratic fitting of MIMIC III distribution of positives above threshold to Rabin distribution of positives above threshold presented in Figure S1. We used linear (once quadratic) functions to artificially raise the number of points in every bin of distribution of positives, then estimated Rabin precision by probability curves. The analysis of distributions, shown in Figure S2, proved useful in restoring the increased shape for models without operational features, slightly less so with them but this is negligible as in practice operational features are unavailable.

| Probability interval |  | 0.1 | 0.2 | 0.3 | 0.4 | 0.5 | 0.6 | 0.7 | 0.8 | 0.9 | 1 |
| --- | --- | --- | --- | --- | --- | --- | --- | --- | --- | --- | --- |
| MIMIC with  operational features | positives | 597 | 178 | 77 | 42 | 24 | 27 | 20 | 15 | 25 | 20 |
|  | negatives | 20250 | 874 | 268 | 85 | 37 | 10 | 15 | 3 | 2 | 0 |
|  |  |  |  |  |  |  |  |  |  |  |  |
| MIMIC without  operational features | positives | 648 | 89 | 40 | 17 | 9 | 7 | 13 | 13 | 15 | 17 |
|  | negatives | 20836 | 520 | 111 | 33 | 35 | 14 | 11 | 3 | 2 | 1 |
|  |  |  |  |  |  |  |  |  |  |  |  |
| Adaptation with  operational features | positives | 149 | 26 | 7 | 2 | 1 | 0 | 0 | 0 | 0 | 0 |
|  | negatives | 9522 | 228 | 89 | 37 | 23 | 14 | 6 | 4 | 1 | 0 |
|  |  |  |  |  |  |  |  |  |  |  |  |
| Adaptation without  operational features | positives | 127 | 25 | 5 | 8 | 5 | 4 | 2 | 0 | 0 | 0 |
|  | negatives | 8495 | 290 | 121 | 58 | 49 | 30 | 12 | 8 | 2 | 0 |
|  |  |  |  |  |  |  |  |  |  |  |  |
| Self with  operational features | positives | 123 | 37 | 10 | 8 | 1 | 1 | 0 | 0 | 0 | 0 |
|  | negatives | 9292 | 375 | 181 | 57 | 14 | 5 | 1 | 0 | 0 | 0 |
|  |  |  |  |  |  |  |  |  |  |  |  |
| Self without | positives | 138 | 26 | 3 | 8 | 3 | 1 | 0 | 0 | 0 | 0 |
| operational features | negatives | 9488 | 250 | 110 | 59 | 13 | 3 | 1 | 0 | 0 | 0 |

**Table S1**. Number of points observed above the threshold for the different models

| **Shape of positives against Shape of positives** | **correlation coefficient** |
| --- | --- |
| Rabin adapt without operational features-MIMIC III without operational features | 0.997 |
| Rabin adapt with operational features-MIMIC III with operational features | 0.989 |
| Rabin Self without operational features-MIMIC III without operational features | 0.991 |
| Rabin Self with operational features-MIMIC III with operational features | 0.998 |

**Table S2**. Correlation coefficients between the distribution shapes of positives above the threshold.

number of positives MIMIC model with operational features

number of positives adapted model with operational features

(a)

number of positives MIMIC model without operational features

number of positives adapted model without operational features

(b)

number of positives MIMIC model with operational features

number of positives self model with operational features

(c)

number of positives MIMIC model without operational features

number of positives self model without operational features

(d)

**Figure S1.** Fit distributions of positives above the threshold.

Precision


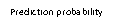


(a)

Precision

Prediction probability

(b)

Prediction probability

Precision

(c)

Precision

Prediction probability

(d)

Figure S2. Approximation of recall by probability distributions.
