# Supplementary material for: A novel machine learning model to predict respiratory failure and invasive mechanical ventilation in critically ill patients suffering from COVID-19
Source: Sci Rep. 2022 Jun 22;12:10573. doi: 10.1038/s41598-022-14758-x (PMC9216294; doi:10.1038/s41598-022-14758-x)
Supplement: Supplementary file 12 — Supplementary Information 12. [file 41598_2022_14758_MOESM12_ESM.docx]

**Supplement 12.** Comparison of the area under the curve (AUC), sensitivity and specificity with and without oversampling

|  |  | MIMIC III | | Adapted MIMIC to Rabin | | Rabin Self | |
| --- | --- | --- | --- | --- | --- | --- | --- |
|  |  | with operational | without operational | with operational | without operational | with operational | without operational |
| Unbalanced | AUC | 0.91 | 0.83 | 0.97 | 0.94 | 0.95 | 0.94 |
|  | Sensitivity | 0.83 | 0.68 | 1.0 | 0.95 | 0.97 | 0.95 |
|  | Specificity | 0.80 | 0.79 | 0.8 | 0.82 | 0.8 | 0.82 |
| RandomOversampling | AUC | 0.90 | 0.83 | 0.96 | 0.92 | 0.95 | 0.92 |
|  | Sensitivity | 0.82 | 0.68 | 0.99 | 0.95 | 0.95 | 0.90 |
|  | Specificity | 0.80 | 0.80 | 0.80 | 0.82 | 0.82 | 0.82 |
| SMOTE | AUC | 0.86 | 0.73 | 0.94 | 0.92 | 0.94 | 0.94 |
|  | Sensitivity | 0.73 | 0.54 | 0.90 | 0.86 | 0.96 | 0.92 |
|  | Specificity | 0.80 | 0.80 | 0.82 | 0.80 | 0.8 | 0.81 |

**Table S1.** Comparison of AUC, sensitivity and specificity with and without oversampling. The comparison was done for the same conditions detailed in Figure 5.

Sensitivity (sometimes named the detection rate in a clinical setting) of a test is the proportion of people who test positive for the disease among those who have the disease.

Specificity of a test is the proportion of those who truly do not have the condition who test negative for the condition.
